# Associations between Vascular Health Indices and Serum Total, Free and Bioavailable 25-Hydroxyvitamin D in Adolescents

**DOI:** 10.1371/journal.pone.0114689

**Published:** 2014-12-05

**Authors:** Ambika P. Ashraf, Jessica A. Alvarez, Tanja Dudenbostel, David Calhoun, Russell Griffin, Xudong Wang, Lynae J. Hanks, Barbara A. Gower

**Affiliations:** 1 Department of Pediatrics, University of Alabama at Birmingham, Birmingham, Alabama, United States of America; 2 Department of Medicine, Emory University School of Medicine, Atlanta, Georgia, United States of America; 3 Cardiovascular Disease, University of Alabama at Birmingham, Birmingham, Alabama, United States of America; 4 Department of Epidemiology, University of Alabama at Birmingham, Birmingham, Alabama, United States of America; 5 Department of Nutrition Sciences, University of Alabama at Birmingham, Birmingham, Alabama, United States of America; Hormel Institute, University of Minnesota, United States of America

## Abstract

**Objective:**

The role of vitamin D in cardiovascular health remains debated as results have been inconsistent. Previous studies have not considered the bioavailability of 25-hydroxy vitamin D [25(OH)D]. Objectives of our study were to investigate the association between serum concentrations of total, free and bioavailable 25(OH)D and independent predictors of cardiovascular risk such as flow mediated dilatation (FMD) and augmentation index (AIx).

**Design:**

This cross-sectional study included 47 post-menarchal, adolescent females [31 African American (AA) and 16 European American (EA)].

**Methods:**

AIx was standardized to a heart rate of 75 beats/min (AIx75). Free and bioavailable 25(OH)D concentrations were calculated from standard formulas.

**Results and Conclusions:**

Mean age of the participants was 15.8±1.4 years and mean body mass index was 23.1±4.0 kg/m^2^. Serum total 25(OH)D was not associated with FMD, but was positively associated with AIx75 in the adjusted model (rho = 0.4, *P* = 0.03). AIx75 was positively associated with bioavailable 25(OH)D (rho = 0.4, *P* = 0.004) and free 25(OH)D (rho = 0.4, *P* = 0.009) and the associations persisted after adjusting for covariates. In race-specific analyses, total, free and bioavailable 25(OH)D were strongly positively associated with AIx75 in AA (rho = 0.5, 0.4, 0.4, respectively), which persisted even after adjusting for covariates. Whereas in EA there was an inverse association between total 25(OH)D and AIx75 in EA (rho = −0.6), which attenuated after adjusting for covariates.

**Conclusion:**

Circulating total, free and bioavailable 25(OH)D were associated with arterial stiffness in adolescent girls, and these associations were race dependent. Notwithstanding, the implications of associations between vascular function indices and 25(OH)D remains unclear.

## Introduction

Vascular stiffness is considered a sub-clinical marker of early vascular damage, as seen in hypertension and atherosclerosis [1], [2]. Many cross-sectional [3]–[5] and limited interventional [6]–[8] studies have previously indicated that vitamin D may play a role in vascular health as indicated by associations with measures such as flow mediated dilatation (FMD), augmentation index (AIx) and pulse wave velocity (PWV). In addition, there have been inconsistent reports on the role of vitamin D in regulating hypertension [9], [10]. The reported role of vitamin D in vascular health has not been supported by all studies [11]. Studies to-date have included mostly non-homogeneous subject populations and used total serum 25-hydroxy vitamin D [25(OH)D] as an indicator of vitamin D status. We hypothesize that the disparate results stem from a lack of accounting for variations in circulating vitamin D binding protein (VDBP), which determines the bioavailability of 25(OH)D.

African Americans (AA) have a higher prevalence of hypertension, greater arterial stiffness, endothelial dysfunction, and insulin resistance compared to European Americans (EA), which has been linked to the widely prevalent vitamin D deficiency among AA [12]–[16]. We previously suggested that lower serum 25(OH)D among AA adults relative to EA adults partially accounts for the racial differences in their vascular function measures [3]. We did not, however, take into account the bioavailability of 25(OH)D. AA reportedly have lower levels of VDBP resulting in comparable concentrations of bioavailable 25(OH)D to whites [17], [18]. Recently, it has been reported that bioavailable vitamin D is better linked to markers of bone mineral metabolism [19]. As adolescence is a period of accelerated metabolic changes, including insulin resistance [20], which we have shown to correlate with VDBP in children [17], it is possible that free and bioavailable 25(OH)D measures are better biomarkers to delineate potential associations with vascular outcomes during adolescence, especially when the subjects include both AA and EA.

Brachial artery FMD is reflective of endothelial function [7], [21]. Central pulse wave or contour analysis is used to generate an AIx, a surrogate measure of arterial stiffness [22]. Differences in regional stiffness as indicated by the stiffness of peripheral muscular arteries such as radial, brachial and femoral and centrally elastic arteries such as carotid and aorta has been previously reported in children with diabetes [23], [24]. The Atherosclerosis Risk in Communities (ARIC) Study demonstrated that incident hypertension was predicted in those in the highest tertile of arterial stiffness at baseline [1]. Early prevention and control of arterial stiffness prior to development of hypertension may reduce cardiovascular morbidity and mortality significantly.

The role of vitamin D in vascular health has not been fully elucidated, particularly among adolescents. Evidence suggests that the atherosclerotic process begins in youth, with subclinical signs of vascular compromise i.e., elevated blood pressure, decreased endothelial elasticity and increased arterial stiffness appearing in adolescence [25]. Previous studies in adults have reported associations between serum 25(OH)D and indices of vascular function in adults. As vitamin D deficiency is widely prevalent among female adolescents [26] and the associations of cardiovascular parameters with vitamin D may be unique in the pediatric population. Therefore, we sought to identify associations between vascular health indices and markers of vitamin D status. Objectives of our study were to investigate the associations of serum concentrations of total, free and bioavailable 25(OH)D with independent predictors of cardiovascular risk such as endothelial dysfunction as assessed by FMD and arterial stiffness as indicated by AIx. We hypothesized that free and bioavailable 25(OH)D measures would have stronger associations with vascular outcomes during adolescence when compared to total serum 25(OH)D. We further hypothesized that total, free and bioavailable 25(OH)D would be inversely associated with AIx (i.e., higher 25(OH)D, less stiffness), and positively associated with FMD (i.e., higher 25(OH)D, increased FMD).

## Subjects and Methods

### Participants

Participants were 47 post-menarchal, female adolescents between ages 14–18. The data were collected between January 2010 and September 2011. Race (European American - EA, African American - AA) was self-defined. Subjects with diabetes, inflammatory disorders, hypertension, smoking, pregnancy in the past year, lactose intolerance, non-ambulatory subjects, those on medications known to influence body composition, glucose metabolism or vascular reactivity (monophasic oral contraceptives, hormonal intrauterine devices, injectable contraceptives, hormone patches, anti-hypertensive, lipid-lowering medications and steroids) and those on vitamin D supplementation were excluded. Adolescents were chosen for the study due to the high prevalence of hypovitaminosis D and insulin resistance in this age group [26]. Only subjects who were post-menarchal and in Tanner stage ≥4 for breast and pubic hair development were included in the study. All tests were conducted in the first 10 days of the menstrual cycle (follicular stage). All research was approved by the University of Alabama at Birmingham (UAB) Institutional Review Board for Human Use. Written informed consent and assent were obtained before entry to the study.

### Protocol

All subjects had anthropometric measurements (weight, height, and waist circumference), seated blood pressure (BP) and body composition assessments. Waist circumference was measured around the narrowest portion of the torso with a flexible tape measure (Gulick II; County Technology, Inc., Gays Mills, WI). Percentage body fat (% fat) was measured with dual energy X-ray absorptiometry (iDXA, GE-LUNAR Radiation Corp., Madison, WI) with participants lying in the supine position with their arms at their sides. Blood samples were obtained after a 12-hr fast, and serum was stored at −80°C until ready for analysis.

Subjects also completed socioeconomic status (SES) and physical activity questionnaires. SES was measured using the Hollingshead Four-Factor Index of Social Status [27]. Physical activity was measured with the validated Physical Activity Questionnaire (PAQ); a higher score indicates greater reported physical activity [28].

### Vascular Outcomes

Vascular function testing was performed by a single physician who was blinded to participant vitamin D status. Supine BP was measured using the auscultatory method, with the arm properly supported and using the correct cuff size after participants rested for at least 5 minutes in the supine position. BP was measured in both arms, and the average of two readings in the arm with the higher blood pressure reading was used.

Flow-mediated dilation was performed as previously described [3]. FMD was measured non-invasively via high-resolution ultrasound with a 7.5 MHz linear-array probe (Philips HP Agilent Sonos 5500, Andover, MA) after a 30 min rest in the supine position in a quiet, air-conditioned room according to standard guidelines [29]. FMD was defined as the percentage increase in diameter from baseline to peak dilation after ischemia and reactive hyperemia induced by inflation of a BP cuff around the forearm to 50 mm Hg above the subject's supine systolic blood pressure (SBP) for 5 min followed by rapid deflation.

Augmentation index (AIx) was computed from the radial artery waveform using a transfer function (Sphygmocor applanation tonometry system,AtCor Medical, Sydney, Australia) according to guidelines [30]. Participants were in the supine position and rested for at least 10 min. Pulse wave assessments were performed during the same early morning session (7:00–9:00 am) after overnight fasting. Three measurements were acquired and the average/median of both parameters was calculated. AIx is influenced by both central stiffness and peripheral wave reflections and is a considered a mixed measure of central and peripheral arterial stiffness [24]. AIx was standardized to a heart rate of 75 beats/min (AIx75) and a higher A1x indicates stiffer vessels.

### Serum analysis

Serum 25(OH)D was measured using liquid chromatography mass spectrometry (Quest Diagnostics, San Juan Capistrano, CA). Intra-assay and inter-assay coefficient of variation (CV) was 9.92% and 1.25%. Serum PTH was commercially assessed by a two-site immunoradiometric assay (Intra and inter-assay CV was 5.11% and 4.39%, at Quest Diagnostics Nichols Institute, San Juan Capistrano, California). Serum VDBP was measured in duplicate in thawed serum samples by using the R&D Systems Human Vitamin D Binding Protein Quantikine ELISA kit (R&D Systems, Minneapolis, MN,) according to the manufacturer's instructions. Interassay CV was 4.7% at a concentration of 160 mg/ml. Free 25(OH)D and bioavailable 25(OH)D were calculated by previously utilized formulas [17], [19]. Glucose was assayed in 10 µl sera using a Sirrus analyzer (Stanbio, Boerne, TX). The mean intra- and inter-assay coefficients of variation (c.v.) for glucose analysis in the Core Laboratory are 1.28% and 1.53%, respectively. Serum insulin was assayed by immunofluorescence on a TOSOH AIA-II analyzer (TOSOH Corp., South San Francisco, CA); intra-assay CV of 1.5% and inter-assay CV of 4.4%. The homeostatic model assessment of basal insulin resistance (HOMA-IR) was calculated using the formula: HOMA-IR  =  [fasting insulin (µU/ml) × fasting glucose (mmol/L)]/22.5 [31].

### Statistical analyses

Descriptive statistics (mean ± SD) were determined for all variables among all participants and by racial group. To identify the demographic and other indices that were independently associated with VDBP, a general linear model was constructed. A backward stepwise procedure was followed so that those variables with P<0.2 were retained in the model. Simple Spearman correlations were used to explore relationships of total, free, and bioavailable 25(OH)D with vascular outcomes. Vascular outcomes found to be significant or approaching significance were used in subsequent multiple linear regression (MLR) analyses to investigate the relationships between total, free, and bioavailable 25(OH)D and vascular outcomes independent of racial group, age, percent body fat and sequentially adjusted for fasting insulin [3], [17]. Our correlation matrix was chosen based on a priori knowledge from the literature as well as from our studies. We have added height to the model for AIx75 as an inverse relationship exists between AIx and height [32], as height directly influences arterial pressure wave forms and distance of wave reflection sites (used for estimation of AIx). Similarly, height influences BP and hence is included in the model for systolic and diastolic BP (SBP, DBP) [33]. Preliminary analyses indicated potential collinearity between racial group and 25(OH)D; thus, relationships were also analyzed within each racial group using partial Spearman correlations sequentially adjusting for age, percent body fat and fasting insulin (for all), and, also for height (for AIx75, SBP, and DBP). The distributions of studentized residuals for all MLR models were examined. All tests were two-sided and assumed a 5% significance level. With this significance level and sample size of 47, an observed effect size of 0.4 can be observed (*P*<0.05, two-sided). Analyses were performed using SAS software (version 9.1; SAS Institute, Cary, NC).

## Results

The study cohort included 47 post-menarchal, adolescent girls. Descriptive characteristics of all participants combined and within each racial group are shown in Table 1. Participants were generally of normal weight (mean BMI 23.3±4.5 kg/m^2^). AA tended to have greater BMI (24.2 vs. 21.55kg/m^2^,*P* = 0.05) than EA. Fasting insulin and HOMA-IR were significantly higher in AA compared to EA (11.2 vs. 6.6 µIU/ml, *P* = 0.0003 and 2.7 vs. 1.5, 0.0004 respectively). AA had significantly lower total serum 25(OH)D (14.8 vs. 27.9 ng/ml, *P*<0.0001) and VDBP (106.9 vs. 232.1 µg/ml, *P* = <0.0001) compared to EA; but, the free and bioavailable 25(OH)D were similar in both groups. The AIx75 and FMD were not statistically different between races. Reported physical activity or SES did not differ between racial groups (Table 1). The SBP and DBP were statistically significantly different between racial groups, SBP 117.4±8.6 vs. 111.7±6.0 mm Hg, *P* = 0.03 and DBP 70.9±7.8 vs. 65.0±5.2 mm Hg, *P* = 0.01 respectively (this racial differences in SBP and DBP, was attenuated after adjusting for age, percent body fat and fasting insulin, *P* = 0.12 and 0.11 respectively).

**Table 1:** Descriptive characteristics among all participants and by racial group.

Variables	All (n = 47)	AA (n = 31)	EA (n = 16)	*P*_racial group_ *
Age (yr)	15.9±1.4	15.7±1.4	16.2±1.4	0.24
Height (cm)	164.9±7.0	164.2±7.6	166.2±5.7	0.35
Weight (kg)	62.7±12.3	64.413.4	59.5±9.6	0.21
BMI (kg/m^2^)	23.3±4.5	24.2±4.5	21.5±3.9	**0.05**
Waist circumference (cm)	71.8±8.2	73.0±9.1	69.5±5.9	0.18
Percent body fat (%)	25.5±5.5	26.4±5.9	23.6±4.1	0.11
SES total score	41.9±11.1	41.2±9.7	43.2±13.7	0.57
Physical activity score (PAQ)	2.1±0.7	2.0±0.7	2.2±0.7	0.5
Supine SBP (mm Hg)	115.6±8.2	117.4±8.6	111.7±6.0	**0.03**
Supine DBP (mm Hg)	69.1±7.6	70.9±7.8	65.0±5.2	**0.01**
Heart rate (beats/min)	64.6±10.5	66.1±10.6	61.2±98.9	0.14
FMD (%)	10.5±4.9	10.0±5.0	11.3±4.7	0.40
AIx75 (%)	−0.9±9.8	0.9±9.6	−4.8±9.4	0.07
PTH (pg/ml)	38.0±16.6	40.4±18.0	33.5±12.5	0.18
Total 25(OH)D (ng/ml)	19.3±8.7	14.8±5.7	27.9±6.7	**<0.0001**
VDBP (µg/ml)	149.5±77.3	106.9±48.7	232.1±50.2	**<0.0001**
Free 25(OH)D (pg/ml)	9.7±4	9.9±4	9.2±3	0.56
Bioavailable 25(OH)D (ng/ml)	3.4±1.3	3.5±1.5	3.3±1.0	0.59
Fasting insulin (µIU/ml)	9.6±5.4	11.2±5.9	6.6±2.0	**0.0003**
HOMA-IR	2.3±1.4	2.7±1.5	1.5±0.5	**0.0004**

*As measured by two-sample independent t-test.

Bolded values represent those that are significantly different between racial groups (*P*≤0.05).

Abbreviations: AA, African American; EA, European American; BMI, body mass index; SES, socioeconomic status; SBP, systolic blood pressure; DBP, diastolic blood pressure; FMD, flow-mediated dilation; AIx75, augmentation index standardized to a heart rate of 75 beats/min; PTH, parathyroid hormone; 25(OH)D, 25-hydroxyvitamin D; VDBP, vitamin D binding protein; HOMA-IR, homeostatic model assessment of insulin resistance.

We further analyzed associations between vascular function indices and demographic/metabolic variables to identify potential covariates. FMD was inversely associated with percent body fat (r = −0.3, P = 0.03) and diastolic blood pressure (r = −0.3, *P* = 0.03). AIx 75 was inversely associated with BMI (r = −0.4, *P* = 0.02) and percent body fat (r = −0.4, *P* = 0.03)- see table 2. Bivariate analyses suggested that race, age total 25(OH)D were significantly associated with VDBP (see Table 3). However, the reduced multivariate model using stepwise backward regression indicated that only race (*P*<0.0001) and age (*P* = 0.0005) were significant predictors of VDBP. After controlling for age, race and fasting insulin, total 25(OH)D was no longer a significant predictor of VDBP.

**Table 2:** Spearman correlations [rho (*P*-value)] of vascular function indices with demographic and metabolic variables.

Variables	FMD		AIx75	
	Crude	Partial*	Crude	Partial*
BMI (kg/m^2^)	−0.23 (0.12)	−0.28 (0.08)	−0.21 (0.17)	−**0.37 (0.02)**
Age	0.07 (0.66)	0.06 (0.70)	0.04 (0.79)	0.10 (0.52)
Height	−0.26 (0.07)	−0.28 (0.06)	−**0.41 (0.005)**	−**0.40 (0.01)**
Percent body fat (%)	**−0.35 (0.02)**	**−0.33 (0.03)**	−0.23 (0.13)	−**0.35 (0.03**)
Supine SBP (mm Hg)	−0.05 (0.77)	0.05 (0.77)	−0.11 (0.46)	−0.25 (0.11)
Supine DBP (mm Hg)	**−0.32 (0.03)**	**−0.34 (0.03**)	**0.30 (0.04)**	0.10 (0.53)
Fasting insulin (µIU/ml)	−0.09 (0.53)	−0.09 (0.6)	0.15 (0.33)	−0.02 (0.9)
HOMA-IR	−0.03 (0.83)	0.00 (0.99)	0.12 (0.44)	−0.11 (0.48)
PTH (pg/ml)	−0.19 (0.21)	−0.09 (0.56)	−0.09 (0.57)	−0.10 (0.55)
VDBP (µg/ml)	−0.00 (0.99)	−0.18 (0.27)	−0.21 (0.16)	−0.07 (0.64)

*Correlations adjusting for race.

Bolded values represent those that are statistically significant (*P*≤0.05).

Abbreviations: BMI, body mass index; SBP, systolic blood pressure; DBP, diastolic blood pressure; HOMA-IR, homeostatic model assessment of insulin resistance; PTH, parathyroid hormone; VDBP, vitamin D binding protein; FMD, flow-mediated dilation; AIx75, augmentation index standardized to a heart rate of 75 beats/min.

**Table 3:** Multiple linear regression analyses of variables modifying VDBP concentrations.

	Crude		Adjusted 1		Adjusted 2	
Variables	β	*P*	β	*P*	β	*P*
Race	−125.27	**<0.0001**	−110.97	**<0.0001**	−116.31	**<0.0001**
Age	−241.77	**0.048**	20.08	**0.0002**	17.57	**0.0005**
% body fat	1.06	0.60	–	–	–	–
PTH	−0.71	0.30	–	–	–	–
Total 25(OH)D	5.09	**<.0001**	0.27	0.81		
Fasting glucose	−1.18	0.38	–	–	–	–
Fasting insulin	−5.92	0.006	−0.13	0.93	–	–

Legend: General linear models using backward stepwise regression used to obtain β estimates and *P*-values. Bolded values represent those that are statistically significant (*P* ≤0.05). Abbreviations: PTH, parathyroid hormone; 25(OH)D, 25-hydroxyvitamin D; VDBP, vitamin D binding protein.

Spearman correlation analyses between vitamin D measures and vascular measures are illustrated in (Table 4). Total, free and bioavailable 25(OH)D were not associated with SBP or DBP. FMD was not associated with concentration of total or free 25(OH)D; but was positively associated with bioavailable 25(OH)D (rho = 0.3, *P* = 0.04). The association attenuated after adjusting for age, race and percent body fat (rho = 0.28, *P* = 0.08) and after sequentially adjusting for fasting insulin (rho = 0.28, P = 0.08).

**Table 4:** Crude and partial Spearman correlations for the association between 25(OH)D measures and vascular outcomes.

	Total 25(OH)D			Free 25(OH)D			Bioavailable 25(OH)D		
Variables	Crude	Partial*	Partial‡	Crude	Partial*	Partial‡	Crude	Partial*	Partial‡
AIx75	0.07	0.35	0.36	0.38	0.34	0.35	0.42	0.37	0.39
	*P* = 0.67	***P*** ** = 0.03**	***P*** ** = 0.03**	***P*** ** = 0.009**	***P*** ** = 0.04**	***P*** ** = 0.03**	***P*** ** = 0.004**	***P*** ** = 0.02**	***P*** ** = 0.02**
FMD	0.23	0.10	0.10	0.27	0.25	0.25	0.30	0.28	0.28
	*P* = 0.13	*P* = 0.55	*P* = 0.55	*P* = 0.07	*P* = 0.11	*P* = 0.12	***P*** ** = 0.04**	*P* = 0.08	*P* = 0.08
Supine SBP	−0.25	−0.04	−0.04	−0.12	−0.19	−0.20	−0.10	−0.18	−0.19
	*P* = 0.09	*P* = 0.81	*P* = 0.81	*P* = 0.43	*P* = 0.24	*P* = 0.24	*P* = 0.5	*P* = 0.27	*P* = 0.27
Supine DBP	−0.11	0.23	0.21	0.23	0.15	0.14	0.23	0.18	0.17
	*P* = 0.46	*P* = 0.17	*P* = 0.21	*P* = 0.13	*P* = 0.35	*P* = 0.40	*P* = 0.13	*P* = 0.27	*P* = 0.33

*All correlations after adjusting for age, race and percent body fat.

‡ Correlations also adjusted fasting insulin for all outcomes (i.e., AIx75, FMD, SBP and DBP), and, also adjusted for height for outcome variables AIx75, SBP, DBP.

Bolded values represent those that are statistically significant (*P*≤0.05). Abbreviations: 25(OH)D, 25-hydroxyvitamin D; AIx75, augmentation index standardized to a heart rate of 75 beats/min; FMD, flow-mediated dilation; SBP, systolic blood pressure; DBP, diastolic blood pressure.

The correlation between A1x75 and total serum 25(OH)D was statistically significant in the adjusted model (rho = 0.4, *P* = 0.03). AIx75 was also positively associated with bioavailable 25(OH)D (rho = 0.4, *P* = 0.004) and free 25(OH)D (rho = 0.4, P = 0.009). The associations persisted after adjusting for age, race and percent body fat, fasting insulin and height (bioavailable 25(OH)D: rho = 0.4, P = 0.02 and free 25(OH)D: rho = 0.4, P = 0.03).

When analyzed by racial groups, total, free and bioavailable 25(OH)D were strongly positively associated with AIx75 in AA (rho = 0.5, 0.4, 0.4, respectively) after adjusting for age, height, and percent body fat (Table 5). After further adjusting for fasting insulin, the associations persisted in AA [total 25(OH)D vs A1x75 rho = 0.5, *P* = 0.01, free 25(OH)D vs A1x75 rho = 0.5, *P* = 0.01, bioavailable 25(OH)D vs A1x75 rho = 0.4, *P* = 03]. Even though total 25(OH)D was inversely associated with AIx75 in EA (rho = −0.6), it attenuated after adjusting for covariates and, furthermore, the associations between AIx75 and free and bioavailable 25(OH)D were not statistically significant. Total, free and bioavailable 25(OH)D were not associated with FMD in race-specific analyses (i.e., AA only or EA only).

**Table 5:** Partial Spearman correlations for the association between 25(OH)D measures and vascular outcomes by race.

	African-American						European American					
	Total 25(OH)D		Free 25(OH)D		Bioavailable 25(OH)D		Total 25(OH)D		Free 25(OH)D		Bioavailable 25(OH)D	
Variables	Partial*	Partial‡	Partial*	Partial‡	Partial*	Partial‡	Partial*	Partial‡	Partial*	Partial‡	Partial*	Partial‡
AIx75	0.51 ***P*** ** = 0.008**	0.51 ***P*** ** = 0.01**	0.48 ***P*** ** = 0.01**	0.48 ***P*** ** = 0.02**	0.44 ***P*** ** = 0.02**	0.44 ***P*** ** = 0.03**	−0.63 ***P*** ** = 0.05**	−0.61 *P* = 0.08	−0.42 *P* = 0.23	−0.42 *P* = 0.26	−0.29 *P* = 0.42	−0.30 *P* = 0.43
FMD	0.17 *P* = 0.40	0.17 *P* = 0.41	0.23 *P* = 0.24	0.23 *P* = 0.26	0.22 *P* = 0.27	0.22 *P* = 0.28	−0.01 *P* = 0.97	−0.02 *P* = 0.95	0.22 *P* = 0.52	0.23 *P* = 0.48	0.32 *P* = 0.34	0.33 *P* = 0.30
Supine SBP	−0.08 *P* = 0.70	−0.08 *P* = 0.72	−0.33 *P* = 0.10	−0.32 *P* = 0.11	−0.30 *P* = 0.13	−0.30 *P* = 0.14	0.08 *P* = 0.83	0.15 *P* = 0.69	0.03 *P* = 0.95	0.05 *P* = 0.90	0.03 *P* = 0.93	0.03 *P* = 0.94
Supine DBP	0.27 *P* = 0.18	0.26 *P* = 0.21	0.22 *P* = 0.29	0.21 *P* = 0.31	0.26 *P* = 0.20	0.25 *P* = 0.23	0.02 *P* = 0.95	−0.05 *P* = 0.90	0.02 *P* = 0.96	−0.004 *P* = 0.99	−0.06 *P* = 0.87	−0.06 *P* = 0.88

*All correlations adjusted for percent body fat and age. For AIx75, SBP and DBP, correlations also adjusted for height.

‡ Correlations further adjusted for fasting insulin.

Bolded values represent those that are statistically significant (*P*≤0.05). Abbreviations: 25(OH)D, 25-hydroxyvitamin D; AIx75, augmentation index standardized to a heart rate of 75 beats/min; FMD, flow-mediated dilatation; SBP, systolic blood pressure; DBP, diastolic blood pressure.

## Discussion

In this study we found that serum total, free and bioavailable 25(OH)D were associated with arterial stiffness (AIx75) in adolescent girls. However, in contrast to the reported inverse associations between 25(OH)D and AIx75 in adults we found positive associations between circulating 25(OH)D metabolites and AIx75. Moreover, these associations were race-dependent with AA demonstrating positive associations and EA showing inverse yet non-significant associations. This study also re-emphasizes the fact that there are no significant racial differences in bioavailable and free 25(OH)D between AA and EA [18]. The apparent lower serum total 25(OH)D in AA is annulled when VDBP is taken into account.

There are several potential mechanisms by which vitamin D could influence vascular function measures [3], [8], [34]. Vitamin D receptors and 1α-hydoxylase enzyme are present on the heart and blood vessels smooth muscle and endothelial cells. Calcitriol reduces parathyroid hormone, influences endothelial and vascular smooth-muscle cell function and controls inflammation. Calcitriol also down regulates the renin-angiotensin-aldosterone system, in addition to regulating cell proliferation. Vitamin D is known to reduce oxidative stress and attenuate inflammation.

AA adolescents generally have higher systolic and diastolic BP relative to EA. However, these differences appear to be influenced to an extent by circulating insulin levels, as this difference was attenuated after adjusting for fasting insulin. The fact that endothelial dysfunction and arterial stiffness are early, sub-clinical markers of vascular damage and are a prelude to hypertension [1], [11], may explain the lack of associations between vitamin D metabolites and systolic/diastolic BP, despite independent associations of 25(OH)D with AIx, in our cohort of healthy, relatively normal weight adolescents.

In contrast to the reported findings in adults [3], [35], FMD and AIx 75 were not different between AA and EA adolescents, suggesting potential factors appearing after puberty that predispose to the race-related differences in arterial stiffness and endothelial function. An intriguing study result was the positive associations between total, free and bioavailable 25(OH)D and AIx75; the implications of which are not clear. The unexpected positive relationship in our study between AIx75 and vitamin D biomarkers may be a reflection of a developmental phenomenon of adolescence. AIx75 reflects a combination of both central/large artery stiffness (i.e., PWV) and magnitude of peripheral arterial wave reflections [24]. Peripheral arteries are more amplified compared to the central arteries, during this time period [36]. We postulate that the positive association between free and bioavailable 25(OH)D to AIx75 in adolescents may indicate an effect of vitamin D on the magnitude of arterial wave reflections (but not on arterial stiffness, per se).

Further analyses revealed that the positive association between vitamin D measures and AIx75 was unique to AA alone. Although we did not have sufficient sample size to show a significant association in EA, there was clearly different directionality between racial groups. This adds to the questions raised by a recent study suggesting low total 25(OH)D blood levels were linked to greater risk of incident coronary heart disease in whites, but not in blacks [37]. Another large study from the National Health and Nutrition Examination Survey (NHANES III) also failed to show associations between low serum 25(OH)D concentration and all-cause mortality among non-Hispanic black participants [38]. The potential for a detrimental effect of higher levels of bioavailable 25(OH)D to vascular function among AA adolescents cannot be discounted for reason(s) not yet understood. Dong et al have previously demonstrated that the PWV in black youth improves after supplementation with 2000 IU of vitamin D for 16 weeks [8]. It is plausible that the positive association between free and bioavailable 25(OH)D to AIx 75 may indicate a deleterious effect of higher vitamin D concentrations on arterial wave reflections (but, not on PWV) in AA. Future investigations are warranted in order to evaluate the reason for this finding, including validating use of AIx75 among adolescents and race-specificity of AIx75 and vitamin D measures.

Another salient observation was that age and race were independent predictors of VDBP among the variables studied. The VDBP levels may have stronger impact during adolescence compared to adult life as age is a determinant of VDBP [17]. We found that either total, free or bioavailable 25(OH)D measurements are able to delineate the associations between vitamin D and vascular function indices in adolescence, if we adjust for confounders of VDBP. It is possible conflicting results of vitamin D and vascular function studies are due to lack of measurement of free and bioavailable 25(OH)D and/or not taking into account factors that are highly determinant of VDBP levels.

The main limitations of our study are its cross sectional nature, lack of direct measurement of large artery stiffness (PWV) and small sample size (particularly in race-stratified analysis). At the time of the study the transfer function for deriving the aortic waveform (for pulse wave analysis) had only been validated in adults (18 years of age or older), with a very low prevalence of vascular abnormalities reported even in those with high risk conditions such as type 1 diabetes [23], [24]. Only clinical trials using vitamin D supplementation utilizing multiple age and racial groups could definitely evaluate the true cause-effect relationship between vitamin D and AIx75. Efforts to infer true associations between vitamin D status and changes in cardiovascular parameters will require longitudinal studies which utilize multiple age and racial groups. In addition, although the AIx and FMD are considered subclinical markers of disease, the clinical importance of these measures in terms of cardiovascular outcomes is not definitive. Even though all subjects were studied in the follicular phase of their menstrual cycle, we did not measure serum estrogen concentrations on day 3 of the follicular phase. Dietary recall information was not available for the data analysis. However, given the inherent limitations of accurate recall and subjective self-reported diet, particularly among female adolescents, and the complexity of sources of vitamin D (i.e., dietary and cutaneous exogenous sources), the objectively measured circulating 25(OH)D most robustly reflect vitamin D status. Inclusion of genetic admixture estimates to delineate racial differences would have added stronger significance to our findings. However, there is a very high reported correlation between parental-reported race of children and composite value of genetic admixture in cohorts representative of a pediatric population within this geographic region [39]. We also acknowledge that there are controversies regarding the formula used to estimate bioavailable 25(OH)D concentrations and the monoclonal antibody assay used herein for VDBP measurement. Potential ethnic differences in the immunoreactivity against variant VDBPs may also exist [40]. However, both issues have been logically addressed by Powe et al [41], including the fact that polyclonal antibodies raised against VDBP may cross-react with other proteins [41]. Nonetheless, availability of direct assay for the measurement of bioavailable 25(OH)D will overcome this limitation and will provide validation to our findings. Future research for identifying the determinants of VDBP is important to answer the questions related to bioavailability of vitamin D.

The strengths of our study include a homogeneous population of post-menarchal adolescent females, both racial groups, and, measurement of AIx which has not been previously addressed in relation to vitamin D in teens. Although we cannot substantiate mechanistic reasons in this cross-sectional analysis, vitamin D intervention studies could delineate true cause-effect relationships.

## Conclusions

In spite of very low total 25(OH)D concentrations, AA have similar bioavailable 25(OH)D concentrations compared to EA, when taking into account of the VDBP, which influences its bioavailability. This cross-sectional study suggests a positive association between vitamin D level and AIx75, which may be specific to AA adolescents, the implications of which are not clear. Long-term, randomized, placebo-controlled trials investigating race-dependent effects of vitamin D supplementation on vascular function among adolescents are warranted.

## Supporting Information

S1 File Data spreadsheet containing all variables analyzed.
(XLS)
